# Intravenous Immunoglobulin-Induced Aseptic Meningitis in a Dermatomyositis Patient

**DOI:** 10.7759/cureus.58242

**Published:** 2024-04-14

**Authors:** Leah Rosoph, Luisa Ladel, Ronak Patel, Patrick Webster

**Affiliations:** 1 Internal Medicine, Yale University School of Medicine, Internal Medicine Residency Program at Norwalk Hospital, Norwalk, USA; 2 Rheumatology, Norwalk Hospital, Norwalk, USA

**Keywords:** drug-induced meningitis, aseptic meningitis, infusion reaction, intravenous immunoglobulins (ivig), autoimmune, dermatomyositis

## Abstract

Aseptic meningitis is a rare but serious complication of treatment with intravenous immunoglobulin (IVIG) and often mimics meningitis of infectious etiology which poses a challenge for timely diagnosis. Although there are published recommendations on the management of IVIG-induced complications, there are no clear guidelines on the continuation of IVIG use after resolution of aseptic meningitis. We present a case of IVIG-induced aseptic meningitis in a patient with a history of refractory dermatomyositis who had been treated with immunosuppressive therapy and IVIG infusions for over a year. The patient developed intense head and neck pain with associated photophobia 24 hours after the most recent IVIG infusion. The patient was managed with supportive care consisting of intravenous fluids and analgesics. The patient’s aseptic meningitis resolved without neurological complications. Ultimately, the patient was restarted on IVIG due to the recurrence of weakness from dermatomyositis. The patient tolerated re-initiation of IVIG without recurrence of IVIG-induced complications. This case highlights the importance of considering IVIG-induced aseptic meningitis as a differential diagnosis in evaluating patients with non-infectious meningitis even after regular IVIG infusions. This case also demonstrates that it is safe to reinitiate IVIG after the resolution of IVIG-induced aseptic meningitis.

## Introduction

Meningitis, referring to the inflammation of the meninges of the brain and spinal cord, is generally differentiated based on cerebrospinal fluid (CSF) analysis from lumbar punctures, specifically based on findings of bacterial cultures. In 1924, aseptic meningitis syndrome was first described by Wallgren, who characterized acute meningitis associated with a negative CSF Gram stain and culture, lacking systemic illness or parameningeal focus [1]. Aseptic meningitis can be further differentiated into infectious, such as viral or fungal, vs. non-infectious etiologies, including systemic inflammatory disease, neoplastic, and drug-induced [2]. Drug-induced aseptic meningitis (DIAM) is a diagnosis of exclusion and complicates many diagnoses due to overlapping clinical features, such as neck stiffness and headache [3]. DIAM is an uncommon complication of some commonly prescribed medications, including trimethoprim-sulfamethoxazole, non-steroidal anti-inflammatory drugs (NSAIDs), azathioprine, anti-CD3 monoclonal antibody, and intravenous immunoglobulin (IVIG). The underlying mechanism of action is suspected to be a form of delayed hypersensitivity reaction [4].

IVIG is routinely used to treat neurological, rheumatological, and hematological diseases. Adverse reactions to IVIG are reported in as many as 81% of infusions to as few as 1%. Side effects of IVIG infusion can range from headaches to life-threatening anaphylactic disorders. IVIG-induced aseptic meningitis accounts for as few as 0.067-1% of all infusion-related reactions [5,6]. Most reactions are delayed and occur in patients receiving higher doses (1-2 g/kg body weight) of immunoglobulins [5].

In this study, we report a case of IVIG-induced aseptic meningitis in a 47-year-old woman with a history of dermatomyositis.

## Case presentation

A 47-year-old Hispanic female with a past medical history of diabetes, asthma, hypertension, hyperlipidemia, migraines, and dermatomyositis presented to the emergency department (ED) with a 24-hour history of acute-onset headache, nausea, photophobia, vomiting, and fever post-IVIG infusion.

In brief, the patient was diagnosed with dermatomyositis two years before admission with proximal muscle weakness and classical skin findings, including heliotrope eruption, periorbital edema, and Gottron’s papules on the knees. Further workup revealed positive antinuclear antibodies at a titer of 1:1,280 and with a fine speckled pattern, creatine kinase levels elevated greater than 11,000 U/L, and muscle biopsy findings consistent with dermatomyositis.

At the time of presentation, the patient had been trialed on multiple immunosuppressives such as mycophenolate and rituximab with minimal success. Given her refractory disease, she ultimately required the addition of IVIG infusions for disease control. One month before presentation, the patient was maintained on a regimen of mycophenolate 1.5 g twice daily, prednisone 15 mg daily, and monthly IVIG infusions (2 g/kg dosing). Her last IVIG dose was less than 48 hours before her presentation. The patient had previously tolerated IVIG infusion well for six months without any adverse reactions.

**Figure 1:**
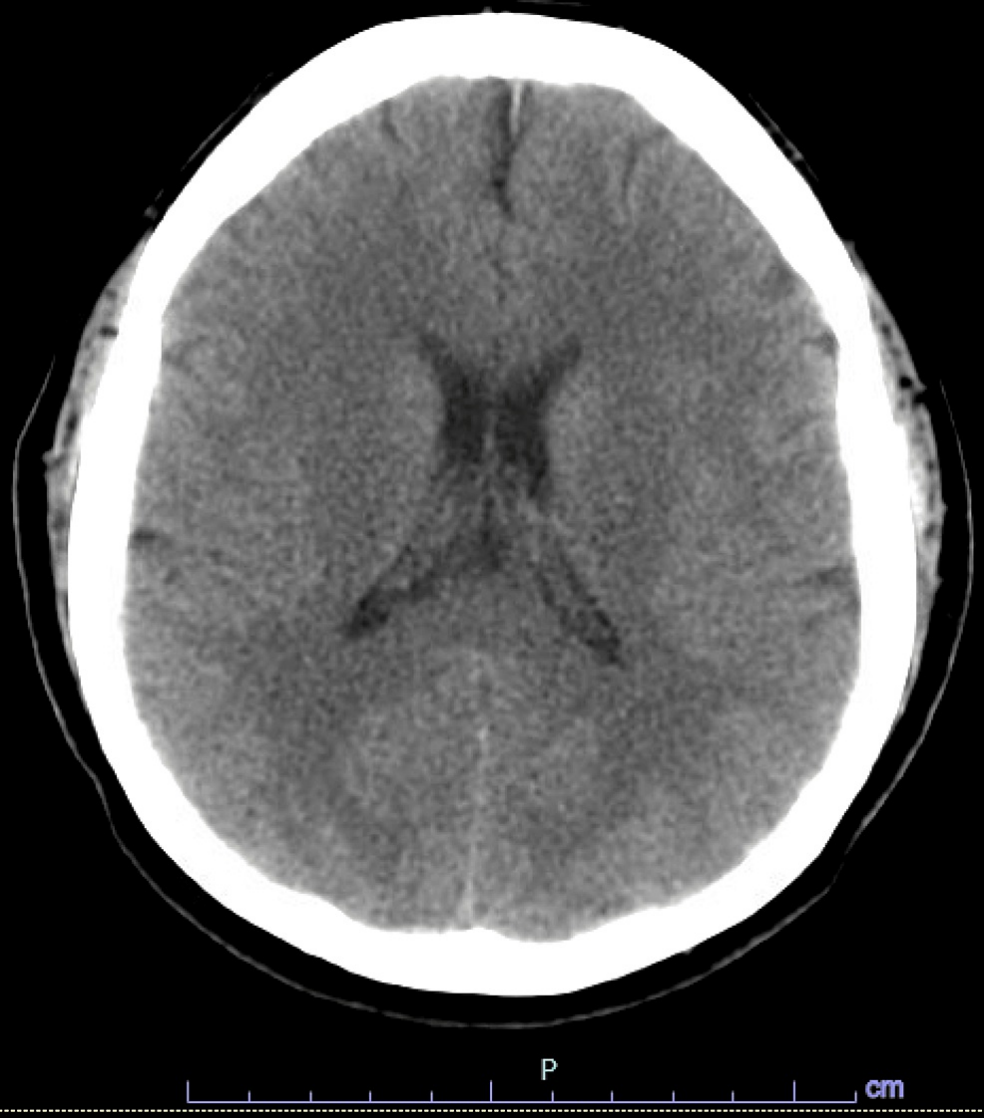
CT of the head and brain, axial view obtained on admission without evidence of intracranial hemorrhage, intracranial infarction, or traumatic brain injury. No mass effect, edema, midline shift, or herniation.

In the ED, the patient was noted to be febrile up to 38.4℃. She reported the sudden onset of a posterior headache that extended to the neck and was sharp in nature associated with nausea and photophobia about 24 hours after her last IVIG infusion. She rated the pain as 10/10 on a visual analog pain scale, which was not relieved with over-the-counter analgesics. On neurological examination, there was no associated focal weakness or changes in sensation to the upper and lower extremities bilaterally. A comprehensive metabolic panel and complete blood count were unremarkable on admission. However, inflammatory markers were elevated, with an erythrocyte sedimentation rate of 64 mm/hour and C-reactive protein of 28.8 mg/L. CT of the head and brain was obtained, which was unremarkable, followed by a lumbar puncture (Figure 1). While waiting for CSF results, she was started on empiric treatment with ceftriaxone, vancomycin, ampicillin, and acyclovir. CSF results later revealed neutrophilic pleocytosis with normal glucose and protein levels (Table 1). Subsequently, antibiotics were discontinued as CSF findings were inconsistent with bacterial meningitis and Gram stain was negative. The patient was continued on acyclovir until the herpes simplex virus and varicella-zoster virus polymerase chain reaction were negative. In addition, the CSF was negative for cryptococcus, varicella, herpes simplex, West Nile virus, Powassan virus, arbovirus, California encephalitis, enterovirus, Eastern Equine encephalitis, and Western Equine encephalitis.

**Table 1:** Cerebrospinal fluid analysis on admission.

Cerebrospinal fluid parameter (units)	Patient values		Reference ranges
	Tube 1	Tube 2	
Appearance	Clear	Clear	Clear
Color	Colorless	Colorless	Colorless
Red blood cells (cells per cubic millimeter)	49	2	0
White blood cells (cells per cubic millimeter)	16	26	0–5
Polymorphonuclear (%)	91	89	2 ± 5
Lymphocytes (%)	9	9	62 ± 34
Monocytes (%)	0	2	36 ± 20
Glucose (mg/dL)	Not reported	62	40–70
Protein (mg/dL)	Not reported	23	15–45

**Figure 2:**
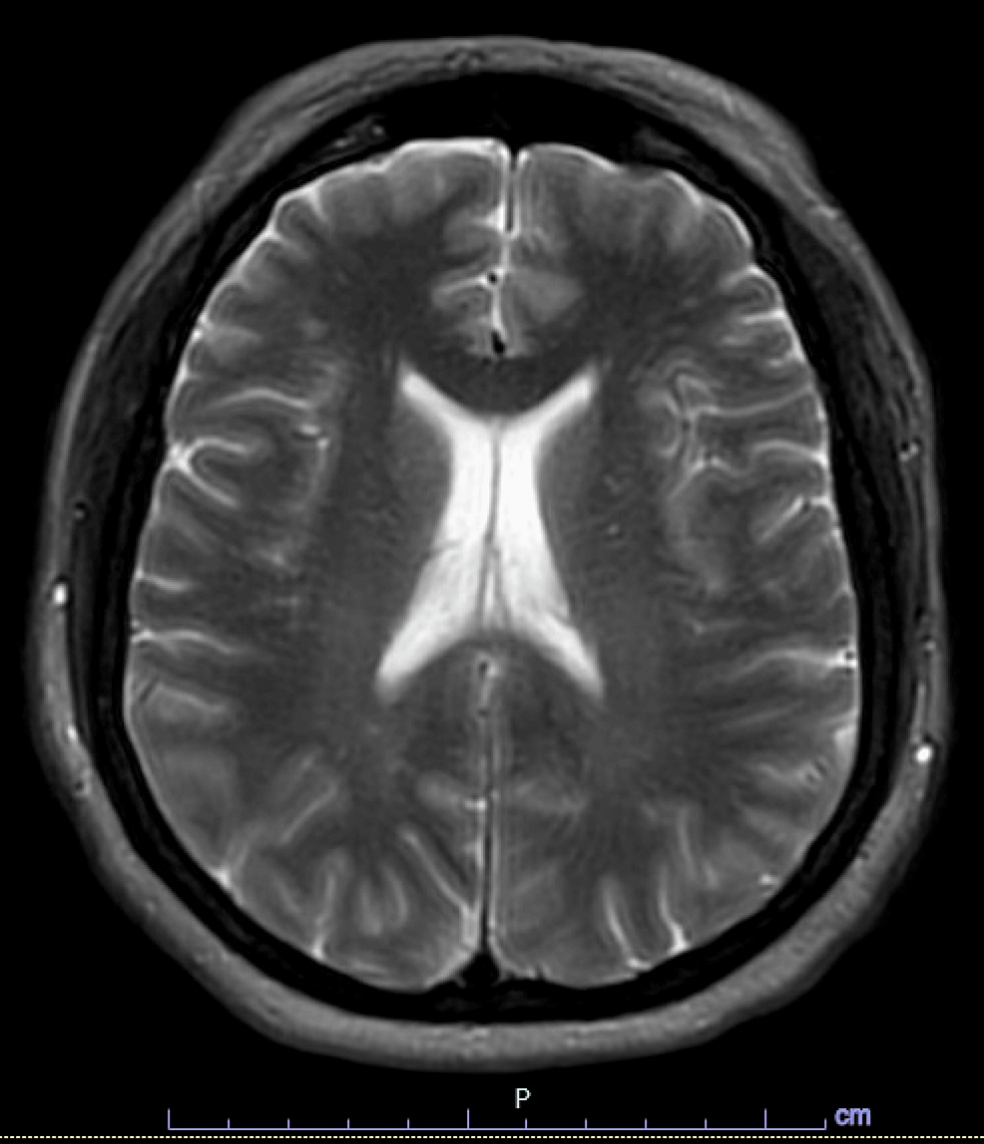
MRI of the brain with and without contrast, axial view notable for scattered subcortical fluid-attenuated inversion recovery hyperintensities.

The patient’s clinical course was complicated by the development of paresthesia on the right side of her face in the V1 to V3 distribution about 48 hours after the first symptom onset. MRI was negative for acute infarct, hemorrhage, or contrast enhancement. There were scattered dominantly subcortical fluid-attenuated inversion recovery hyperintensities suggestive of multiple etiologies, including infection versus inflammation (Figure 2). The patient’s headaches and paresthesia gradually improved over the course of four days with supportive care consisting of analgesics and intravenous fluids. A diagnosis of IVIG-induced aseptic meningitis was made given the absence of identifiable infectious etiology and clinical improvement with discontinuation of IVIG. The patient was discharged after four days with complete resolution of presenting symptoms.

The patient was seen in the outpatient setting shortly after discharge and was initially continued on prednisone and mycophenolate mofetil. Approximately one month after discharge, the patient’s muscle strength began to decline with a notable rise in creatine kinase concerning for a dermatomyositis flare. IVIG infusions at 2 g/kg were restarted and given over a three-day duration. Her dosing frequency was decreased from every four weeks to eight weeks. Her infusion rate was lowered to avoid infusion-related adverse effects, including recurrence of aseptic meningitis. The patient has since undergone three cycles of IVIG infusions without complications and with an improvement in her dermatomyositis symptoms.

## Discussion

Dermatomyositis is a rare autoimmune condition characterized by skin findings and progressive proximal muscle weakness. Glucocorticoids are considered the first-line treatment for dermatomyositis. However, in patients who have a suboptimal response or are unable to tolerate steroids, additional immunosuppressive agents are warranted [7].

Our patient was initiated on IVIG due to a suboptimal response to a combination of steroids, rituximab, and mycophenolate mofetil. IVIG first gained popularity for autoimmune conditions after being studied in idiopathic thrombocytopenic purpura in 1981 [8]. Since then, the use of IVIG has been indicated in many rheumatological diseases, such as systemic lupus erythematosus, rheumatoid arthritis, and antineutrophilic cytoplasmic antibody-positive vasculitis [9]. A broad range of adverse effects is associated with IVIG use, which can be divided into two categories, i.e., immediate reactions versus delayed reactions.

The most common immediate reactions are flu-like symptoms seen in as many as 80% of patients [6,10]. This reaction is considered secondary to cytokine effects, as interleukin-6 and tumor necrosis factor-alpha are found in IVIG products [6]. Regarding neurologic adverse effects associated with IVIG, headache is considered the most common, while posterior reversible encephalopathy syndrome, abducens nerve palsy, and aseptic meningitis have been reported as less frequent adverse effects [6]. One of the most serious complications is IVIG-induced aseptic meningitis which is reported in 0.067-1% of cases depending on the underlying disease [2,3]. Although the exact underlying etiology of IVIG-induced aseptic meningitis remains unknown, multiple mechanisms have been proposed, such as a delayed hypersensitivity reaction, direct irritation within the leptomeninges, or inflammatory cytokine release inducing meningeal inflammation [2,11].

There are multiple identified potential risk factors for the development of IVIG-induced aseptic meningitis. The known risk factors are a history of migraines and a high dosage of IVIG (1-2 g/kg body weight), which were both present in our patient [4]. Regardless of the rate of infusion or brand of IVIG, a history of headaches is associated with a higher risk of developing aseptic meningitis [2,5]. First-time infusion, rapid rate of infusion, and prior exposure to blood products have also been found to increase the risk of IVIG-induced aseptic meningitis [5]. Additionally, several studies note the onset of symptoms within 48 hours of initiating the IVIG infusion [4,5].

A large literature review was conducted in 2022 to evaluate the relationship between IVIG treatment and aseptic meningitis [3]. Overall, 44 cases of IVIG-associated aseptic meningitis were reported, with the average age of onset being 22.4 years. Most cases were reported in children [3]. Aseptic meningitis was diagnosed most often in immune thrombocytopenic purpura with only one case reported in dermatomyositis. Overall, 50% of patients evaluated had lymphocyte-predominant CSF findings, whereas our patient had a neutrophilic-predominant CSF [3].

Although IVIG-induced aseptic meningitis is reported in many autoimmune diseases, it is rarely reported in patients with dermatomyositis. To our knowledge, ours is only the third case reported in the English literature. The first known case was a 48-year-old female who received high-dose IVIG for treatment of dermatomyositis between 1990 and 1994 [5]. Her CSF was notable for lymphocyte predominance. The second case was seen in a five-year-old female with juvenile dermatomyositis after receiving a new formulation of high-dose IVIG. Her CSF was neutrophilic predominant [12]. Similar to previously described cases, our patient was of the same sex and received high-dose IVIG.

One of the main reasons IVIG-induced aseptic meningitis is rarely reported in patients with dermatomyositis is that the use of IVIG in dermatomyositis was only recently approved in 2021 based on the ProDERM trial [7,13]. The ProDERM trial was a phase 3 randomized controlled study that evaluated the efficacy of IVIG versus placebo in patients with dermatomyositis. The ProDerm trial found that 79% of patients treated with IVIG compared to 44% of patients who received a placebo had significant improvement in symptoms associated with dermatomyositis. Clinical response was based on the total improvement score which is based on six criteria, including muscle strength, physicians’ global assessment of disease activity, patients’ assessment of disease activity, the health assessment questionnaire, extramuscular disease activity, and serum muscle enzyme levels (creatine kinase, lactate dehydrogenase, aldolase, alanine aminotransferase, and aspartate aminotransferase) [7]. Headache was the most reported adverse effect of IVIG in this study, although there were no reported cases of aseptic meningitis [7]. The trial had many limitations, including the number of patients enrolled (n = 95), a short study duration of 16 weeks, and the exclusion of cancer-associated and amyopathic dermatomyositis cases. The study concluded that IVIG was associated with at least minimal improvement at week 16 when compared to placebo; however, IVIG was associated with a higher risk of thromboembolic events. As the treatment for dermatomyositis with IVIG becomes more common, we can expect an increase in cases of dermatomyositis-related IVIG-induced aseptic meningitis. Thus, our patient’s presentation could serve as an important predictor of future aseptic meningitis cases.

There are no strict guidelines for the management of IVIG-induced aseptic meningitis [14]. Bacterial meningitis should first be excluded with lumbar puncture and CSF culture. Once bacterial meningitis is ruled out, symptomatic treatment with fluids, antipyretics, and analgesics is crucial in managing aseptic meningitis [15-17]. Although difficult to diagnose, if there is a high suspicion of DIAM (negative viral polymerase chain reaction and negative cerebrospinal fluid culture), immediate cessation of the offending agent is recommended [15,18]. Notably, IVIG-induced aseptic meningitis does not necessarily warrant cessation of IVIG treatment, as this has been studied in patients with Guillain-Barré syndrome who developed IVIG-induced aseptic meningitis [4]. However, appropriate measures should be taken to avoid the recurrence of aseptic meningitis with future IVIG treatments. Most studies suggest that the best way to lower the risk is to reduce the infusion rate [3,14,19-21]. Based on a study published in 2018 by Guo et al. that reviewed all adverse effects associated with IVIG, the authors initially recommended slowing the infusion rate to no more than 1 mL/minute during the first 30 minutes to no more than 3 mL/minute after the first 30 minutes [14]. There appears to be a direct correlation between the rate of infusion and the severity of adverse effects [3]. Of note, slowing the rate of infusion only reduced the incidence of aseptic meningitis, but did not avoid it entirely [3]. In addition, patients could be pre-medicated with antihistamines, NSAIDs, or corticosteroids and should be pre-hydrated with normal saline to reduce adverse reactions [3,14]. Antihistamines are believed to be particularly useful given aseptic meningitis may develop secondary to a hypersensitivity reaction [3]. Furthermore, switching to subcutaneous forms of IVIG as well as modifying the immunoglobulin preparation altogether have also been proposed [3,22]. A study by Racosta et al. noted that the relative risk of moderate to systemic adverse effects was 28% lower in patients who received subcutaneous immunoglobulin rather than IVIG [23]. Further studies need to be conducted to specifically investigate the management of IVIG-induced aseptic meningitis in dermatomyositis as best management and prevention strategies remain controversial. Future investigations might also shed light on potential predictive tools that could aid physicians in risk assessment and patient education before beginning IVIG treatment.

## Conclusions

The long-term outcomes and potential sequelae of the continuation of IVIG use after recovering from IVIG-induced aseptic meningitis remain largely unknown. In this case, the continuation of IVIG after recovery demonstrated improvement in the underlying disorder of dermatomyositis without recurrence of aseptic meningitis after four months of continued IVIG use. Therefore, while clinicians should be aware of this significant complication of IVIG treatment, we highlight that it should not deter from continued use if necessary to maintain disease control. More research is needed to further evaluate the pathophysiology, management, and prevention of IVIG-induced aseptic meningitis in patients with dermatomyositis.
